# Syndecan-1 Is Overexpressed in Human Thoracic Aneurysm but Is Dispensable for the Disease Progression in a Mouse Model

**DOI:** 10.3389/fcvm.2022.839743

**Published:** 2022-04-25

**Authors:** Sara Zalghout, Sophie Vo, Véronique Arocas, Soumaya Jadoui, Eva Hamade, Bassam Badran, Olivier Oudar, Nathalie Charnaux, Dan Longrois, Yacine Boulaftali, Marie-Christine Bouton, Benjamin Richard

**Affiliations:** ^1^LVTS, INSERM, U1148, Paris, France; ^2^Université Sorbonne Paris Nord, Villetaneuse, France; ^3^Laboratory of Cancer Biology and Molecular Immunology, Faculty of Sciences-I, Lebanese University, Beirut, Lebanon; ^4^Université Sorbonne Paris Nord, Bobigny, France; ^5^Université de Paris, Paris, France

**Keywords:** Syndecan-1, aneurysm, proteoglycan, extracellular matrix, smooth muscle cell (SMC)

## Abstract

Glycosaminoglycans (GAGs) pooling has long been considered as one of the histopathological characteristics defining thoracic aortic aneurysm (TAA) together with smooth muscle cells (SMCs) apoptosis and elastin fibers degradation. However, little information is known about GAGs composition or their potential implication in TAA pathology. Syndecan-1 (SDC-1) is a heparan sulfate proteoglycan that is implicated in extracellular matrix (ECM) interaction and assembly, regulation of SMCs phenotype, and various aspects of inflammation in the vascular wall. Therefore, the aim of this study was to determine whether SDC-1 expression was regulated in human TAA and to analyze its role in a mouse model of this disease. In the current work, the regulation of SDC-1 was examined in human biopsies by RT-qPCR, ELISA, and immunohistochemistry. In addition, the role of SDC-1 was evaluated in descending TAA *in vivo* using a mouse model combining both aortic wall weakening and hypertension. Our results showed that both SDC-1 mRNA and protein are overexpressed in the media layer of human TAA specimens. RT-qPCR experiments revealed a 3.6-fold overexpression of SDC-1 mRNA (*p* = 0.0024) and ELISA assays showed that SDC-1 protein was increased 2.3 times in TAA samples compared with healthy counterparts (221 ± 24 vs. 96 ± 33 pg/mg of tissue, respectively, *p* = 0.0012). Immunofluorescence imaging provided evidence that SMCs are the major cell type expressing SDC-1 in TAA media. Similarly, in the mouse model used, SDC-1 expression was increased in TAA specimens compared to healthy samples. Although its protective role against abdominal aneurysm has been reported, we observed that SDC-1 was dispensable for TAA prevalence or rupture. In addition, SDC-1 deficiency did not alter the extent of aortic wall dilatation, elastin degradation, collagen deposition, or leukocyte recruitment in our TAA model. These findings suggest that SDC-1 could be a biomarker revealing TAA pathology. Future investigations could uncover the underlying mechanisms leading to regulation of SDC-1 expression in TAA.

## Introduction

The mortality rate due to thoracic aortic aneurysm (TAA) rupture is in considerable increase and the effectiveness of current drug therapies is still limited and controversial (1–3). Glycosaminoglycans (GAGs) pooling has long been considered as one of the histopathological characteristics defining TAAs, together with smooth muscle cells (SMCs) apoptosis or loss of contractility and elastin fibers degradation (4–6). However, the identity of the accumulated GAGs in TAA and their potential involvement in the disease are poorly studied. A better understanding of the molecular changes occurring in TAA pathophysiology may open up new avenues for its treatment.

Even though proteoglycans (PGs; i.e. core protein with attached GAGs) occupy 1 to 5% mass fraction of a healthy arterial wall, they are crucial contributors to the aortic structural integrity and functioning and in particular to its mechanical homeostasis (7). PGs accumulation may exert a swelling pressure on the elastic laminae, resulting in their separation and a consequent modification of the wall mechanical properties, giving rise to a weakened wall and participating in TAA dissections and ruptures (7, 8). Recently, it has been reported that aggrecan and versican, two chondroitin sulfate PGs, accumulate in regions of medial degeneration in ascending TAAs and dissections and may contribute to extracellular matrix (ECM) disruption (9). In addition, the levels of heparan and chondroitin sulfate PGs are increased after vascular injury and in dissected aortas (10–12).

Syndecan-1 (SDC-1), a transmembrane heparan sulfate PG, is involved in various aspects of inflammatory diseases (13–15), wound healing (16), and ECM components' interactions or assembly (17, 18). SDC-1 has been shown to maintain a differentiated (19) and a contractile state of SMCs (12). In addition, it mediates cell-cell and cell-matrix interactions by acting as a co-receptor for binding to ECM molecules or growth factors such as fibronectin or VEGF, respectively (20, 21). Therefore, SDC-1 is hypothesized to play a role in the development of aortic wall pathologies.

SDC-1 has indeed been shown, in two mice models of abdominal aortic aneurysm (AAA) in which aneurysm was induced by elastase or angiotensin II (Ang II) infusion (on Apo E deficient background) (22), to exert a protective role against AAA development by attenuating the inflammatory response and reducing protease activity (22). In contrast, no data are available regarding its role in TAA development.

In the current study, we investigated SDC-1 expression in the aortic wall of patients with TAA arising from different etiologies. We also examined the disease progression and characteristics by the use of an aneurysm mouse model generated by the combination of aortic wall weakness and hypertension in SDC-1^+/+^ or SDC-1^−/−^ mice on C57Bl/6J background.

## Materials and Methods

### Human Samples

Twenty-five human ascending TAA samples were obtained from patients at the time of prophylactic surgical repair and eleven healthy control thoracic aortic tissues were obtained from deceased organ donors under the authorization of the French Biomedicine Agency (PFS 09-007). The clinical data relative to organ donors (healthy) and patients are presented in Supplementary Table 1. Aortic preparation involved direct paraformaldehyde fixation for histological studies or immediate macroscopic dissection to separate the distinct aortic layers (intima, media, adventitia) followed by direct freezing for RT-qPCR and ELISA analysis.

### Quantification of mRNA and Protein Levels of SDC-1 in Human Samples

Frozen media tissues from healthy or TAA specimens were pulverized using liquid nitrogen freezer mill (6870 SPEX Certiprep 6750). Protein extraction was done by powder lysis in RIPA buffer (20 mg/mL). SDC-1 media protein abundance was assessed using ELISA (R & D systems, DY2780) according to the manufacturer's instructions, each sample being run in duplicate. For RNA extraction, 50 to 100 mg of powder were homogenized in Trizol Reagent (Invitrogen). Total RNA was extracted using RNeasy extraction kit (Qiagen) and 1 μg was reverse transcribed with RT Maxima First Strand kit (K1642, Thermo Scientific). The real time qPCR was performed on cDNA using the StepOnePlus system (Applied Biosystems). Expression levels of mRNA were normalized to hypoxanthine guanine phosphoribosyl transferase (*HPRT1*). Sequences of primers are listed in Table 1. Fold changes of gene expression were calculated using ΔΔCT method.

**Table 1:** Primers used for quantitative polymerase chain reaction (qPCR; human samples).

**Gene**	**Forward primer sequence (5'-3')**	**Reverse primer sequence (5'-3')**
*HPRT1*	TGAGGATTTGGAAAGGGTGT	CCAGCAGGTCAGCAAAGAA
*SDC-1*	GCCAAGCTGACCTTCACAC	CCCAGCACCTCTTTCCTGT

### Histology and Immunostaining

For histological studies, human and murine fixed aortic tissues were embedded in paraffin and sectioned into 7 μm thick sections. Antigen retrieval was done by heating the sections in a water bath at 95°C for 30 min in a DAKO^®^ Target Retrieval Solution, pH 9 (code S2367). Sections were permeabilized by 0.1% Triton-X 100 for 5 min. For DAB IHC, endogenous peroxidase activity was blocked by 3% (vol/vol) H_2_O_2_ for 30 min. Blocking was performed with DAKO^®^ protein block-serum free (code # X0909) for 1 h at room temperature. Sections were incubated overnight at 4°C with primary antibodies (Supplementary Table 2).

For DAB IHC, slides were washed with PBS and treated for 1 h at room temperature with LSAB2 System, HRP kit for human, and Peroxidase AffiniPure donkey anti-rat secondary antibody for mice samples (Supplementary Table 2). The 3′-diaminobenzidine tetra-hydrochloride chromogen (DAB, K3468, DAKO^®^) was added to all sections and the reaction was stopped with distilled water. Counterstaining was done with hematoxylin (for mice IHC). Slides were then mounted with Eukitt® mounting media.

For immunofluorescence studies following primary antibody incubation, sections were treated with the corresponding secondary antibodies (Supplementary Table 2) for 2 h at room temperature. Nuclei were then stained with DAPI aqueous fluoroshield mounting media (Abcam, ab104139). α-SMA, CD45, and Ly-6G markers were used to identify SMCs, leukocytes, and neutrophils, respectively.

Elastin laminae degradation of mice aorta samples was blind-scored by two different observers after orcein (Sigma-Aldrich) staining, using an ascending scale from 0 to 4 as follows: 0, no elastin degradation; 1, 25% degraded; 2, 50% degraded; 3, 75% degraded; and 4, no elastin layer left.

Collagen deposition was detected with Sirius red (RAL Diagnostics) staining, and its quantification was assessed by image J software after exposing the slides to polarized light (Leica microsystems, DMi8).

### Animals and Experimental Procedures

SDC-1^−/−^ mice were a kind gift from Dr. Pyong Woo Park (Harvard Medical School, Boston) and have been backcrossed for at least 10 generations on a C57BL/6J background.

Mice were born and bred in the animal facility. Animals were housed in individually ventilated cages with *ad libitum* access to food and water. Cage temperature was maintained at 21.0 ± 2.0 °C with a relative humidity of 55.0 ± 5.0%. Animals were kept under a 12-hour light/12-hour dark cycle.

Three-week-old SDC-1^+/+^ and SDC-1^−/−^ mice were divided into three groups: (group 1) control group with no treatment (mice were sacrificed at 8 weeks of age), (groups 2 & 3) treated groups including mice receiving daily intraperitoneal injection of 150 mg/kg/day of β-aminopropionitrile fumarate (BAPN, A3134; Sigma Aldrich) for 28 days, then infused subcutaneously with 1 μg/kg/min of angiotensin II (Ang II, A9525, Sigma Aldrich) during 3 (group 2) or 28 days (group 3) using mini-osmotic pumps (Alzet 2004) (**Figure 2A**). At the end of the protocol, mice were anesthetized and sacrificed. Aortas were harvested and rinsed with saline to remove blood. Aortas were cleaned from the surrounding connective and adipose tissues, fixed in 10% formalin for 24 h at 4°C, and stored in 70% ethanol for further investigations. Necropsy was done for mice that died during the course of the experiment and the site of rupture (thoracic or abdominal) was determined based on macroscopic view of hemorrhage location. Aneurysm (TAA or AAA) was defined as ≥ 50% increase in the mean aortic diameter compared to the same control aortic segment from non-treated mice (please check below quantification section).

### Blood Pressure Measurement

Mice blood pressure was measured non-invasively before pump implantation and up to 5 days post-implantation, using the tail cuff system (BP-2000 SERIES II, Blood Pressure Analysis System ^TM^, Visitech Systems). Mice were habituated for a minimum of 5 consecutive days before the recordings were initiated.

### Measurement of Aortic Diameter

For morphometric analysis, images were taken by an EF-S 60 mm macro lens mounted on a DSLR camera (Canon EOS600D) and used to measure the outer diameter of the early descending thoracic aorta using Image J software. The diameter was determined at the site of dilatation from the average of a minimum of three different measurements of the posterior and inferior sides.

### Statistical Analysis

Values are shown as percentage or mean ± standard error of the mean (SEM). Statistical analysis was performed using Prism GraphPad. The non-parametric Mann-Whitney test was used to compare two groups when data did not display a normal distribution. Fisher's exact test was used when comparing two categorical variables. Gehan-Breslow-Wilcoxon test was used to compare the mice survival curves. *p* < 0.05 were considered significant.

## Results

### SDC-1 Expression Is Increased in the Specimens of Aortic Media From Patients With TAA and Is Expressed by SMCs

**Figure 1:**
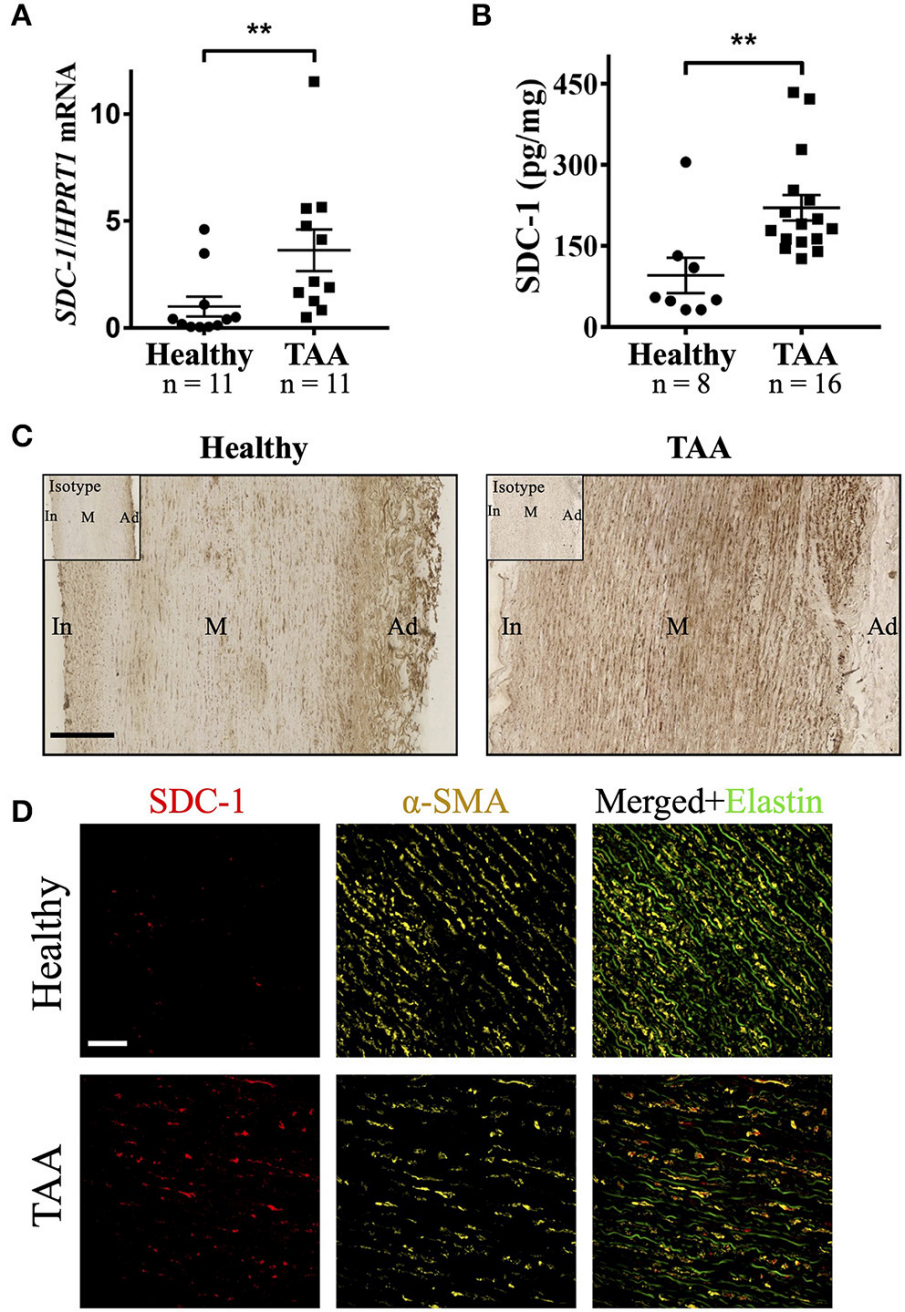
SDC-1 mRNA and protein levels are increased in the media layer of human TAA compared to healthy counterparts and is expressed by SMCs. **(A)** mRNA expression level of SDC-1 in the media layer of healthy aorta and TAA was measured by RT-qPCR and normalized to HPRT1. **(B)** The abundance of SDC-1 protein in human aortic media was assessed by ELISA from healthy or TAA donors. **(C)** Representative images of SDC-1 staining by immunohistochemistry in healthy human and TAA media. Staining with an isotype control was performed as a negative control and shown on the top left of images. Scale bar corresponds to 200 μm. In, intima; M, media; Ad, adventitia. **(D)** Representative immunofluorescence images of SDC-1 (red) and α-SMA (yellow) co-staining in the media of healthy human or TAA media. Green color corresponds to elastin autofluorescence in the merged images. Scale bar corresponds to 50 μm. **(A,B)** Data are presented as mean ± SEM and *p*-values were calculated using non-parametric two-tailed Mann-Whitney test; **p < 0.01.

The expression level of SDC-1 for mRNA and protein was investigated in the medial layer of specimens from human healthy or TAA patients. The mRNA level of SDC-1 in the media of TAA specimens was significantly increased by 3.6 times compared to healthy counterparts (*p* = 0.0024) (Figure 1A). Similarly, a significantly higher protein expression level of SDC-1 was revealed by ELISA in TAA samples (221 ± 24 pg/mg of tissue) compared with healthy (96 ± 33 pg/mg of tissue) human aortic media (*p* = 0.0012) (Figure 1B). This difference was confirmed by IHC (Figure 1C). Immunofluorescence results showed a co-localization between SDC-1 and α-SMA, indicating that most of SDC-1 is expressed by SMCs in human TAA specimens (Figure 1D).

Altogether, these data illustrate that SDC-1 is more expressed at the mRNA and protein levels in TAA media compared to healthy counterparts and SMCs are the cell type overexpressing SDC-1 in TAA media.

### SDC-1 Is Overexpressed in the TAA Developed in the BAPN/Ang II Aneurysm Mouse Model

**Figure 2:**
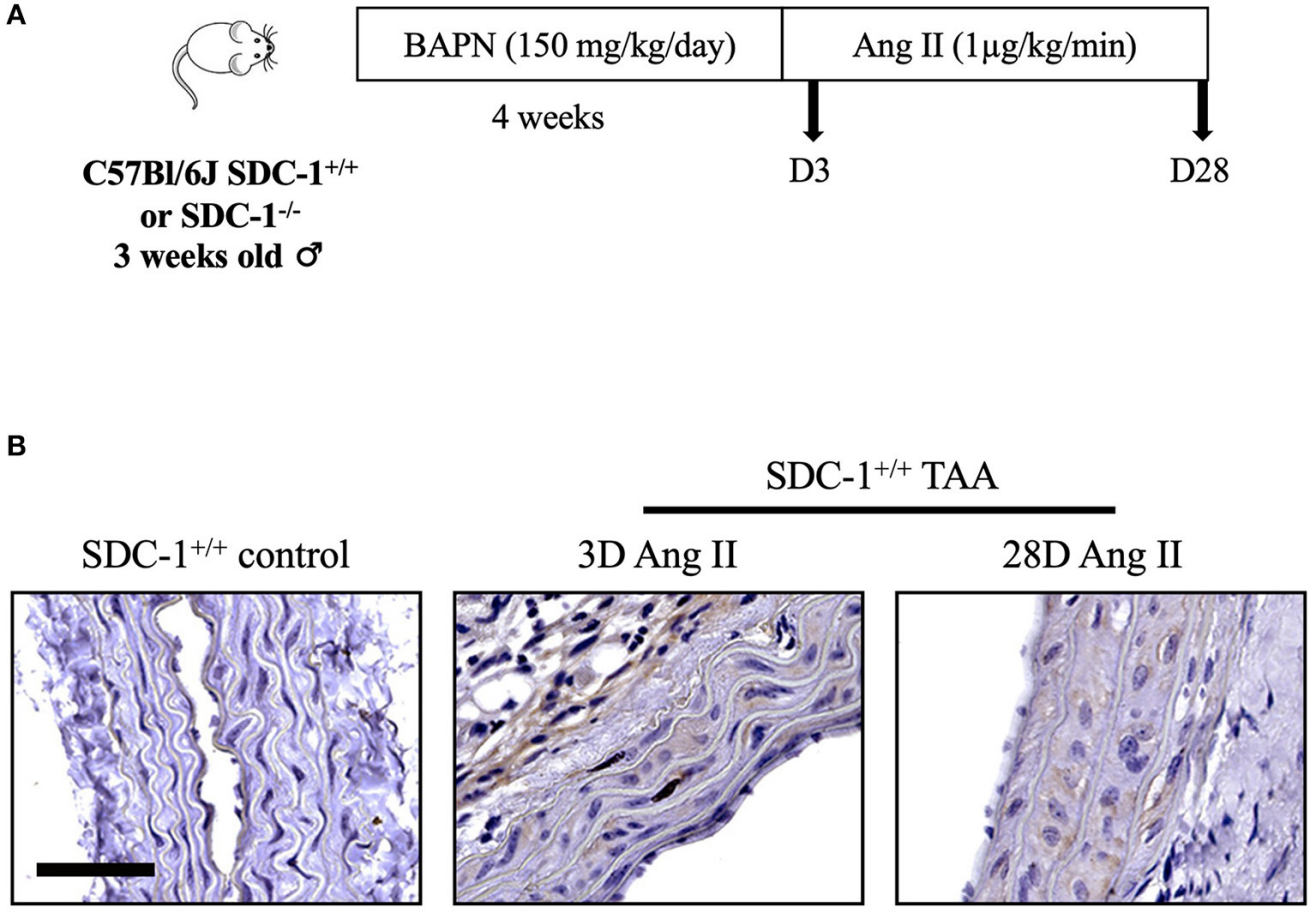
SDC-1 is overexpressed in TAA compared to control aortas in the BAPN/AngII aneurysm mouse model. **(A)** Experimental model used. SDC-1^+/+^ or SDC-1^−/−^ male C57Bl/6J mice at 3 weeks old received intraperitoneal injection of β-amino propionitrile (BAPN) for 4 weeks followed by subcutaneous infusion of angiotensin II (Ang II) by pump implantation for 3 (D3) or 28 (D28) days, then sacrificed. **(B)** Representative images of SDC-1 immunostaining in control (*n* = 3) and TAA specimens after 3 (*n* = 2) or 28 days (*n* = 3) of Ang II infusion. Scale bar corresponds to 50 μm.

The role of SDC-1 on TAA development was investigated in an animal model using BAPN and Ang II, known to induce aortic aneurysms in C57Bl/6J mice (23). In the present model, 3-week-old SDC-1^+/+^ or SDC-1^−/−^ male mice received intraperitoneal injection of BAPN for 4 weeks and then subcutaneous infusion of Ang II by pump implantation. Mice received Ang II for 3 or 28 days to mimic early and late stages of TAA development (Figure 2A). The effect of Ang II on arterial blood pressure was confirmed by the significant increase in systolic blood pressure measured after implantation, with no difference between SDC-1^+/+^ and SDC-1^−/−^ mice (data not shown). The use of this Ang II/BAPN model did not generate aneurysm in all studied animals (discussed in the following section). Analysis of SDC-1 expression by IHC revealed that SDC-1 protein expression level was elevated in TAA compared to control aortas after both 3 and 28 days of Ang II infusion (Figure 2B). SDC-1 expression was specific for the TAA development as SDC-1 was not detected in aortas treated only with BAPN or in aortas that did not develop aneurysm after both BAPN and Ang II treatments (at both time points) (Supplementary Figure 1). Therefore, in accordance with results obtained in human specimens, SDC-1 expression was increased in the TAA generated in this mouse model.

### SDC-1 Is Dispensable for TAA Development and Displays a Potential Protective Role Against AAA Development in Mice

**Figure 3:**
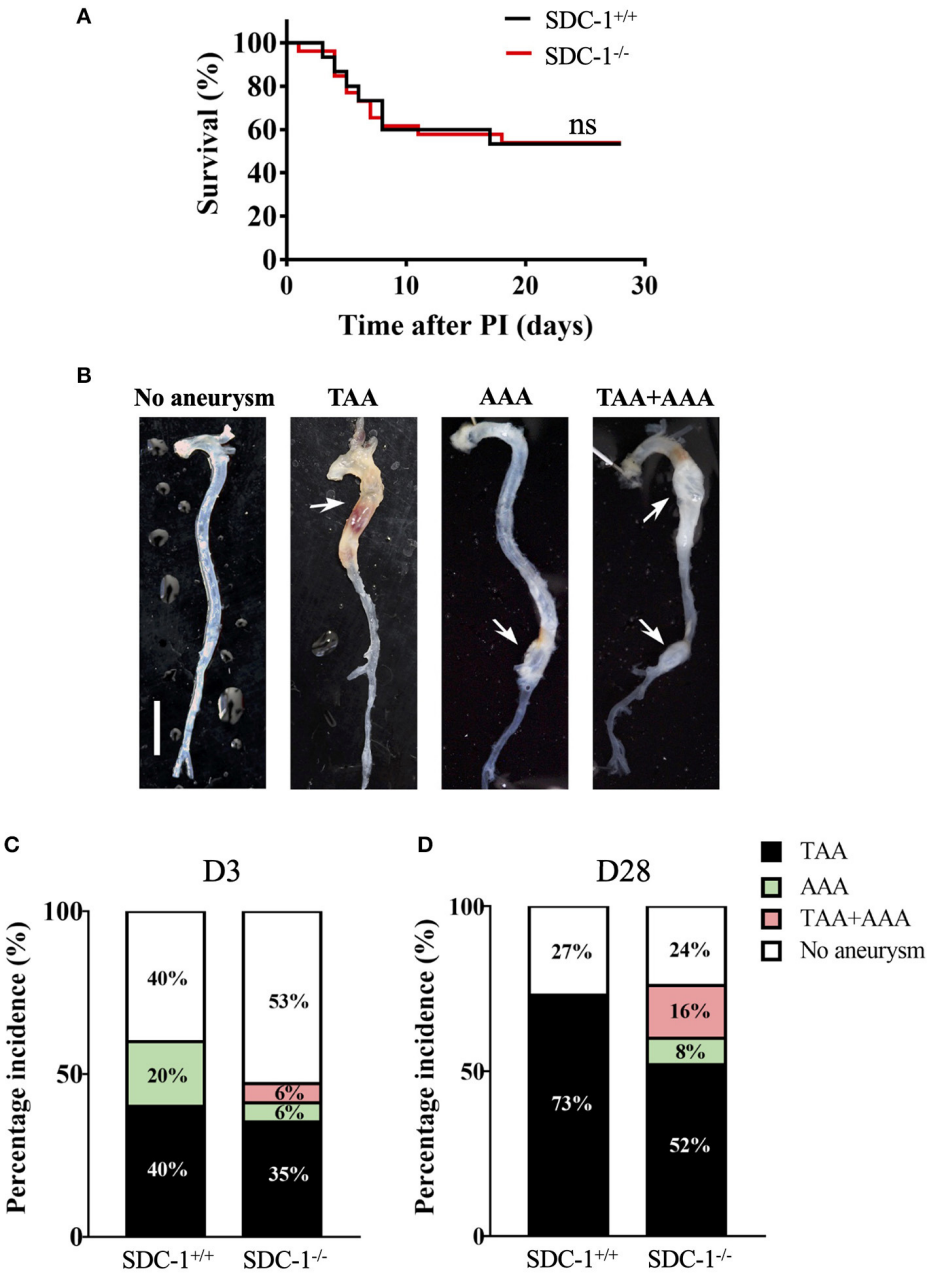
SDC-1 is dispensable for TAA incidence or rupture but tends to protect from AAA development in mice. **(A)** Survival rate of mice after 28 days following Ang II infusion, compared with the Gehan-Breslow-Wilcoxon test. PI, pump implantation; SDC-1^+/+^: n = 15, SDC-1^−/−^: *n* = 26; ns, non-significant. **(B)** Aorta macroscopic images with/without aneurysms 28 days after Ang II infusion. Scale bar corresponds to 5 mm. **(C,D)** Percentage of TAA or AAA incidence in SDC-1^+/+^ or SDC-1^−/−^ mice for 3 **(C)** or 28 days **(D)** of Ang II infusion. **(C)** SDC-1^+/+^: *n* = 5, SDC-1^−/−^: *n* = 17. **(D)** SDC-1^+/+^: *n* = 15, SDC-1^−/−^: *n* = 26.

The survival rate of SDC-1^+/+^ and SDC-1^−/−^ mice was similar during the course of Ang II infusion (Figure 3A), indicating that SDC-1 deficiency did not alter the viability of the mice that developed aneurysm.

As this model is known to induce both thoracic and abdominal aneurysms, TAA and AAA incidence were compared in SDC-1^+/+^ and SDC-1^−/−^ mice. Aortas harvested on day 3 or day 28 from surviving animals were photographed (Figure 3B) and their external diameters were measured (discussed in the following section). After 3 days of Ang II infusion, the proportions of the developed aneurysms were as follows for SDC-1^+/+^ and SDC-1^−/−^ mice respectively: TAA (40 vs. 35%), AAA (20 vs. 6%), and both TAA and AAA (0 vs. 6%) (Figure 3C). These results did not reveal any significant implication of SDC-1 in aneurysm incidence in this mouse model after 3 days of Ang II infusion (Fisher's exact test).

When analyzing aneurysm incidence after 28 days of Ang II infusion, 73% of SDC-1^+/+^ mice developed TAA vs. 52% in SDC-1^−/−^ mice. Moreover, 8% of SDC-1^−/−^ developed AAA whereas none of the SDC-1^+/+^ mice did (Figure 3D). SDC-1^−/−^ mice showed a higher tendency for AAA incidence, alone or in combination with TAA, compared to SDC-1^+/+^ mice, with 6 /26 AAA in SDC-1^−/−^ mice compared to 0/15 AAA in SDC-1^+/+^ mice (*p* = 0.06) (Figure 3D) (Fisher's exact test). These data are in accordance with a previous study reporting a protective effect of SDC-1 against AAA development in a mice model of AAA (22).

### SDC-1 Deficiency Does Not Alter the Extent of Aortic Dilatation, ECM Remodeling, or Leukocytes Recruitment in Descending TAA in Mice

The observation that SDC-1 was not involved in TAA incidence does not rule out the possibility that it could affect the extent of thoracic aortic dilatation or the morphology of the developed TAA.

**Figure 4:**
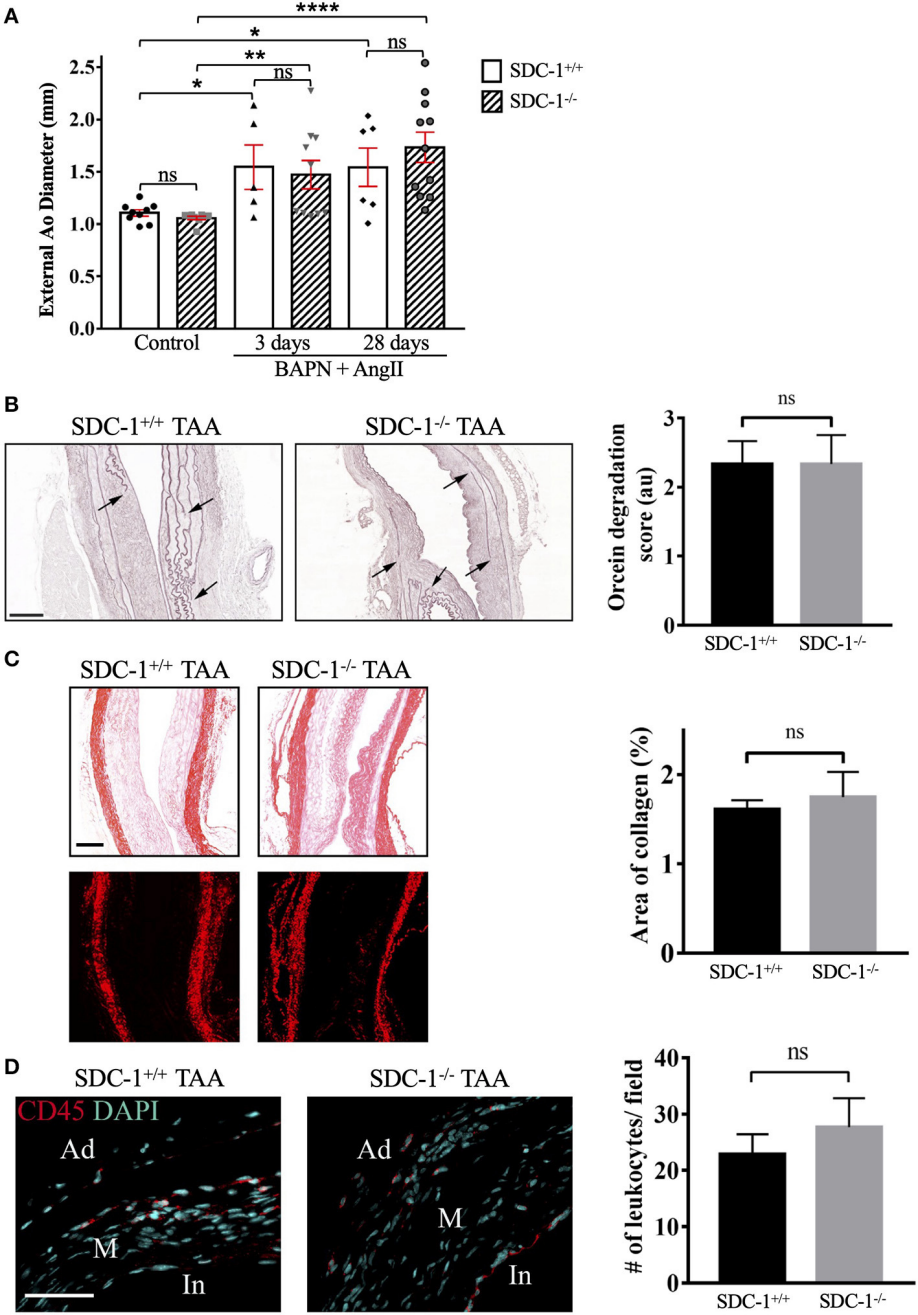
SDC-1 deficiency does not alter the extent of aortic dilatation, ECM remodeling, or leukocytes recruitment in descending TAA in mice. **(A)** Measurement of external descending thoracic diameter in SDC-1^+/+^ and SDC-1^−/−^ mice that received BAPN and Ang II for 3 or 28 days or control (sham) mice. Aorta with a diameter ≥ 1.5 mm was considered as an aorta that developed TAA. SDC-1^+/+^ or SDC-1^−/−^ control: *n* = 9 for both; SDC-1^+/+^ and SDC-1^−/−^ after 3 days: *n* = 5 and *n* = 10, respectively; SDC-1^+/+^ and SDC-1^−/−^ after 28 days: *n* = 6 and *n* = 11, respectively. **(B)** Elastin degradation. Representative images of orcein staining for SDC-1^+/+^ or SDC-1^−/−^ TAA specimens after 28 days following Ang II infusion and quantification of elastin degradation (to the right). Arrows show sites of elastic laminae degradation. SDC-1^+/+^: *n* = 3, SDC-1^−/−^: *n* = 6. Scale bar corresponds to 100 μm. **(C)** Collagen deposition. Representative images of Sirius red staining for SDC-1^+/+^ or SDC-1^−/−^ TAA specimens after 28 days following Ang II infusion visualized by bright-field microscopy (upper images) or polarization microscopy (lower images) and its quantification (to the right) by image J software. Scale bar corresponds to 100 μm. **(D)** CD45 expression. Representative immunofluorescence images of TAA specimens after 28 days of Ang II infusion stained with Ab against CD45 and counterstained with DAPI and its quantification (to the right) by Image J software. In, intima; M, media; Ad, adventitia. Scale bar corresponds to 50 μm. **(C,D)** SDC-1^+/+^: *n* = 3, SDC-1^−/−^: *n* = 7. **(A–D)** Data are presented as mean ± SEM and *p*-values were calculated using non-parametric two-tailed Mann-Whitney test; *p < 0.05, **p < 0.01, ****p < 0.0001, ns: not significant.

Measuring the external diameter of the descending thoracic aorta did not show any difference between SDC-1^+/+^ and SDC-1^−/−^ aortas at both time points (Figure 4A). More specifically, we did not observe any difference in diameter between SDC-1^+/+^ and SDC-1^−/−^ aortas that displayed TAA, 28 days after pump implantation (data not shown).

The ECM remodeling of the developed SDC-1^+/+^ or SDC-1^−/−^ TAA was investigated by assessing the level of elastin degradation and collagen deposition by orcein and Sirius red staining, respectively.

Orcein staining showed that the elastic fibers were disrupted, irregular, and fractured in the TAA tissues of both SDC-1^+/+^ and SDC-1^−/−^ mice (Figure 4B, left). However, no significant difference in the elastin degradation was observed between the two genotypes (Figure 4B, right). Sections stained with Sirius red were examined under bright-field or polarized light microscopy (Figure 4C). Collagen was observed mostly in the adventitia and to a lower extent in the media of SDC-1^+/+^ and SDC-1^−/−^ TAA tissues (Figure 4C, left). Polarized light showed a red color, suggesting that the staining corresponds to collagen type I (24). However, no difference in collagen content was observed between SDC-1^+/+^ and SDC-1^−/−^ TAA specimens (Figure 4C, right).

Increasing evidence supports a role of inflammation or immune cells infiltration in human TAA [reviewed in (25)], and TAA mice models are often associated with inflammation (26, 27). In addition, SDC-1 is involved in various aspects of inflammation such as leukocyte recruitment (28) or its resolution (29). Therefore, leukocyte, and more specifically neutrophil recruitment, were analyzed in SDC-1^+/+^ or SDC-1^−/−^ TAA specimens after 3 (Supplementary Figure 2A) or 28 days (Figure 4D, Supplementary Figure 2B) of Ang II infusion.

Massive recruitment of leukocytes (CD45 staining) and neutrophils (Ly-6G staining) in TAA was observed in a similar manner in both SDC-1^+/+^ and SDC-1^−/−^ mice following 3 days of Ang II infusion (Supplementary Figure 2A). Leukocytes and neutrophils were distributed nearly all over the aorta but sparsely in the intact media. These cells were mainly observed in the adventitia or at the border of the false channel and to a lesser extent in the dissected media part (media around false channel) (Supplementary Figure 2A). Less leukocyte infiltration was detected after 28 days of Ang II infusion compared to 3 days, with still no difference between SDC-1^+/+^ and SDC-1^−/−^ mice (Figure 4D, right and Supplementary Figure 2B). These leukocytes were observed mainly in the intima and adventitia, and to a lesser extent in the media (Figure 4D, left and Supplementary Figure 2B). Moreover, very few neutrophils were observed in the TAA formed in both SDC-1^+/+^ and SDC-1^−/−^ mice at this time point (Supplementary Figure 2B).

Taken together, these results indicate that SDC-1 was not associated with the extent of aortic dilatation, elastin degradation, nor collagen deposition in descending TAA in mice. In addition, SDC-1 was not associated with leukocytes' (and neutrophils') recruitment in descending TAA in this mouse model.

## Discussion

Previous studies suggested that SDC-1 could be an important player in TAA development as it is involved in ECM assembly and organization (18) and SMCs phenotype regulation and mechanosensing (19). The mRNA expression level of SDC-1 (referred previously as syndecan) was previously reported to be increased after vascular injury (30). More recently, an increase of SDC-1 protein expression was observed in the adventitia of human aortas with ascending TAA (31).

In the current study, we report for the first time, by RT-qPCR, ELISA, and histological analyses, that SDC-1 is one of the members of the PGs overexpressed in human TAA medial layer.

Our results also indicate that SMCs are the major cell type expressing SDC-1 in human TAA, which is in line with an *in vitro* study showing that mechanical stress induces the expression of SDC-1 by SMCs (32). This suggests that the altered wall mechanics in TAA induce SDC-1 expression on SMCs. BAPN and Ang II infusion induce vascular remodeling (33), TAA, AAA (23), and thoracic aortic dissection (34). Our model was adapted from a study showing that BAPN and Ang II administration in C57Bl/6J mice without any specific genetic background develop 49% of AAA and 38% of TAA mostly in the ascending part of the aorta (23). However, our SDC-1^+/+^ C57Bl/6J mice did not develop any AAA 28 days after Ang II infusion and almost all of the TAA were found in the descending aorta, at the typical site of B dissection and not distal as found by others (23). The observed differences between these results can be explained by the different age of the mice at the beginning of the experiment (9 vs. 3 weeks), the route of BAPN administration (subcutaneous vs. intraperitoneal), the duration of Ang II treatment (6 vs. 4 weeks), or the mouse background (SDC-1^+/+^ from heterozygous mating vs. commercially available mice).

Our results indicate that SDC-1 is also not involved in the incidence of descending TAA nor in the risk of rupture in our model. However, SDC-1 deficiency has been reported to exacerbate AAA formation and rupture vulnerability in two mice models of abdominal aneurysms (22). We observe similar findings as AAA was observed only in SDC-1^−/−^ mice and not in the SDC-1^+/+^ mice, 28 days after Ang II infusion. The fact that SDC-1 was not involved in the extent of aortic dilatation, elastin degradation, or collagen deposition in TAA in our model suggests that SDC-1 may have a more potent effect on ECM remodeling in abdominal rather than in thoracic aorta, given their different structural and mechanical characteristics (35, 36). The differences in the number of lamellar units, elastin and collagen content, proteinase system, and tension forces contribute to distinctive vascular remodeling at thoracic or abdominal sites [reviewed in (36)]. Moreover, the inflammatory response is amplified in AAA compared to TAA (37), increasing the protease activity and MMPs production.

It is worth mentioning that PGs distribution is heterogeneous throughout the aorta, providing a mechanism for regional-dependent adaptation to variable hemodynamic stress patterns (38). More interestingly, the regulation of PGs has been shown to be distinct in the two types of the disease. For instance, aggrecan and versican accumulate in human ascending TAA (9), whereas proteomic analysis of AAA samples showed reduced abundance of these PGs in comparison to control samples (39). Therefore, it would not be surprising that a single PG performs a distinct function specific to the site of its expression.

Notably, PGs display differential expression depending on the age of the individual (in mice or humans) and the severity of the disease (9, 40–42). Examining the regulation of SDC-1 in early human stages of TAA development is likely unachievable. We assumed that 3 days and 28 days of Ang II infusion corresponded to early and late stages of aneurysm pathophysiology, respectively. However, rupture (late stage of aneurysm) was observed all along the experimental protocol, even 24 h after Ang II infusion. Thus, the used model did not permit us to study a possible contribution of this PG in early stages of the disease.

As observed for the human TAA samples, SDC-1 protein expression increased in mice TAA compared to control aortas at both time points. The observed overexpression in the media, and adventitial layers of TAA specimens 3 days after Ang II infusion should correspond to both SMCs and leukocytes expression. Indeed, 28 days after Ang II infusion, SDC-1 overexpression was observed only in the media and not the adventitia of TAA specimens, in concordance with the observation of decreased leukocytes infiltration at this time point.

We observed an infiltration of neutrophils localized mainly in the adventitia, in borders of the false channel, and in dissected media in TAA specimens 3 days after Ang II infusion, most likely because Ang II, as a vasopressor, is a potent stimulant of neutrophil recruitment (43, 44). Neutrophil infiltration was previously reported to be in the intima following 24 h of Ang II infusion (45). Our data showed that after a longer time of Ang II infusion (3 days), which corresponds to a more advanced stage of aneurysm, neutrophil infiltration took place in the media where dissection was observed and in the adventitia. However, these neutrophils almost disappeared 28 days after Ang II infusion, corresponding possibly to a switch from an acute to a chronic inflammatory phase.

Despite the findings that neutrophil-expressed SDC-1 reduces neutrophils adhesion to the endothelium (15) and that SDC-1 mediates neutrophils resolution by chemokines clearance (29), our data showed that SDC-1 does not play a role in neutrophils recruitment in our model, since no difference was observed between SDC-1^+/+^ or SDC-1^−/−^ TAA specimens.

The impact of pooled PGs/GAGs on medial degeneration could be either a global effect generated by all accumulated PGs, or specific where each PG by itself exerts a particular role during TAA development. A process of compensation (or redundancy) displayed by other PGs expression in the present model could explain the absence of a SDC-1 specific effect, as it has been reported in the context of atherosclerosis between biglycan and perlecan for Apo-B retention (46).

## Conclusion

This study reports an overexpression of SDC-1 in human TAA compared to healthy aortas, suggesting that it can serve as a biomarker for this pathology. The underlying mechanism relevant to this alteration remains to be determined. The mouse model used induced mostly descending TAA, and SDC-1 was not involved in either its incidence nor in its histological characteristics. An implication of SDC-1 in TAA (ascending or descending) could be revealed from studies in other TAA mouse models, possibly associated with ECM genes deficiency as it organizes the ECM and interacts with its proteins. Interestingly, we observed that SDC-1 tends to protect from AAA, in agreement with a previous report (22). Deciphering the protective molecular function of SDC-1 in AAA, and its possible role in TAA, could perhaps aid in the comprehension of its clinical relevance.

## Data Availability Statement

The original contributions presented in the study are included in the article/Supplementary Material, further inquiries can be directed to the corresponding author.

## Ethics Statement

The studies involving human participants were reviewed and approved by INSERM and AP-HP (CEERB du GHU Nord) Institutional Review Board (CPP 05 04 32, Ambroise Paré, Boulogne, France, April in 2005; updated March 2008). The patients/participants provided their written informed consent to participate in this study. The animal study was reviewed and approved by ARRIVE guidelines, Animal Care and Use Committee (2011-14/69-0035).

## Author Contributions

BR conceived and designed the study. SZ performed most of the experiments. BR, SV, and SJ performed some of human and/or mice experiments. M-CB, VA, EH, YB, BR, and SZ analyzed the data. SZ wrote the manuscript with support from BR. M-CB, VA, YB, DL, OO, NC, EH, and BB supervised the research and provided intellectual discussion and editorial advice. All authors contributed to manuscript revision and approved the submitted version.

## Funding

This work was funded by INSERM and Sorbonne Paris Nord University. SZ was funded by the Lebanese University, AZM Saade Association, INSERM, and Sorbonne Paris Nord University.

## Conflict of Interest

The authors declare that the research was conducted in the absence of any commercial or financial relationships that could be construed as a potential conflict of interest.

## Publisher's Note

All claims expressed in this article are solely those of the authors and do not necessarily represent those of their affiliated organizations, or those of the publisher, the editors and the reviewers. Any product that may be evaluated in this article, or claim that may be made by its manufacturer, is not guaranteed or endorsed by the publisher.
