# Supplementary material for: Syndecan-1 Is Overexpressed in Human Thoracic Aneurysm but Is Dispensable for the Disease Progression in a Mouse Model
Source: Front Cardiovasc Med. 2022 Apr 25;9:839743. doi: 10.3389/fcvm.2022.839743 (PMC9082175; doi:10.3389/fcvm.2022.839743)
Supplement: Supplementary file 1 [file Table_1.pdf]

Supplementary table 1:

Table S1: Organ donors' and patients' characteristics. Data are shown as mean ± standard deviation. TAA : Thoracic Aortic Aneurysm

	Organ donors (healthy)	Patients with TAA
n	11	25
age	49.9 ± 9.6	53.9 ± 17.9
Sex, % male	36 %	96 %
