# Supplementary material for: Syndecan-1 Is Overexpressed in Human Thoracic Aneurysm but Is Dispensable for the Disease Progression in a Mouse Model
Source: Front Cardiovasc Med. 2022 Apr 25;9:839743. doi: 10.3389/fcvm.2022.839743 (PMC9082175; doi:10.3389/fcvm.2022.839743)
Supplement: Supplementary file 2 [file Table_2.pdf]

Supplementary table 2:

Table S2: Antibodies used for immunohistochemistry and immunofluorescence experiments

Antigen	Dilution, host	Catalog number	Company
**Primary Ab**			
**Anti-SDC-1 (human IHC)**	10 µg/mL, mouse anti human	Sc-12765	Santacruz
**Anti-SDC-1 (human IF)**	5 µg/mL, rabbit anti human	PA5-16918	Invitrogen
**Anti-α-SMA (human IF)**	0.71 µg/mL mouse anti-human	M0851, clone 1A4	Dako
**Anti-SDC-1 (mice IHC)**	0.5 µg/mL, rat anti mouse	142502	Biolegend
**Anti-Ly-6G**	10 µg/mL, rat anti mouse	551459	BD pharmingen
**Anti-CD45**	8 µg/mL, rabbit anti mouse	ab10558	Abcam
**Secondary Ab /kit**			
**LSAB2 System-HRP (human IHC)**	Goat anti-mouse	KO675	Dako
**Peroxidase AffiniPure (mouse IHC)**	0.8 µg/mL, Donkey anti rat	712-035-153	Jackson ImmunoResearch
**CY3 F(ab')2 anti-rat**	3 µg/mL, goat polyclonal	112-166-062	Interchim
**CY3 F(ab')2 anti-rabbit**	3 µg/mL, goat polyclonal	111-166-045	Interchim
**CY5 F(ab')2 anti-mouse**	3 µg/mL, goat polyclonal	115-606-062D	Interchim
**CY5 F(ab')2 anti-rabbit**	3 µg/mL, goat polyclonal	111-606-144	Interchim
